# Nutrition and Chronobiology as Key Components of Multidisciplinary Therapeutic Interventions for Fibromyalgia and Associated Chronic Fatigue Syndrome: A Narrative and Critical Review

**DOI:** 10.3390/nu16020182

**Published:** 2024-01-05

**Authors:** Noèlia Carrasco-Querol, Lorena Cabricano-Canga, Nerea Bueno Hernández, Alessandra Queiroga Gonçalves, Rosa Caballol Angelats, Macarena Pozo Ariza, Carme Martín-Borràs, Pilar Montesó-Curto, Elisabet Castro Blanco, Maria Rosa Dalmau Llorca, Carina Aguilar Martín

**Affiliations:** 1 Unitat de Suport a la Recerca Terres de l’Ebre, Fundació Institut Universitari per a la Recerca a l’Atenció Primària de Salut Jordi Gol I Gurina (IDIAPJGol), 43500 Tortosa, Spain; nereabhernandez@gmail.com (N.B.H.); aqueiroga@idiapjgol.info (A.Q.G.); amacarena.ebre.ics@gencat.cat (M.P.A.); cmartin@idiapjgol.org (C.M.-B.); ecastro@idiapjgol.info (E.C.B.); caguilar.ebre.ics@gencat.cat (C.A.M.); 2 EAP Dreta Eixample, CAP Roger de Flor, C/Roger de Flor 194, 08013 Barcelona, Spain; lcabricano@eapdretaeixample.cat; 3 Red de Investigación en Cronicidad, Atención Primaria y Promoción de la Salud (RICAPPS), 08007 Barcelona, Spain; 4 Fundació Institut Universitari per a la Recerca a l’Atenció Primària de Salut Jordi Gol I Gurina (IDIAPJGol), 08007 Barcelona, Spain; rcaballol.ebre.ics@gencat.cat (R.C.A.); mpmonteso.ebre.ics@gencat.cat (P.M.-C.); rdalmau.ebre.ics@gencat.cat (M.R.D.L.); 5 Servei d’Atenció Primària Terres de l’Ebre, Institut Català de la Salut (ICS), 43500 Tortosa, Spain; 6 Departament de Fisioteràpia, Facultat de Ciencies de la Salut Blanquerna, Universitat Ramón Llull, 08025 Barcelona, Spain; 7 Departament de Medicina i Cirurgia, Facultat de Medicina i Ciències de la Salut, Universitat Rovira i Virgili (URV), 43201 Reus, Spain; 8 Unitat d’Avaluació i Recerca, Direcció d’Atenció Primària Terres de l’Ebre i Gerència Territorial Terres de l’Ebre, Institut Català de la Salut (ICS), 43500 Tortosa, Spain

**Keywords:** fibromyalgia, chronic fatigue syndrome, nutrition, chronobiology, review, evidence

## Abstract

Fibromyalgia (FM) is often accompanied by chronic fatigue syndrome (CFS). It is a poorly understood disorder that mainly affects women and leads to chronic pain, fatigue, and insomnia, among other symptoms, which decrease quality of life. Due to the inefficiency of current pharmacological treatments, increasing interest is being directed towards non-pharmacological multicomponent therapies. However, nutrition and chronobiology are often overlooked when developing multicomponent therapies. This narrative and critical review explore the relevance of nutritional and chronobiological strategies in the therapeutic management of FM and the often-associated CFS. Reviewed literature offers scientific evidence for the association of dietary habits, nutrient levels, body composition, gut microbiota imbalance, chronobiological alterations, and their interrelation with the development and severity of symptoms. This review highlights the key role of nutrition and chronobiology as relevant and indispensable components in a multidisciplinary approach to FM and CFS.

## 1. Introduction

Fibromyalgia (FM) and associated chronic fatigue syndrome (CFS) are considered central sensitization syndromes that often present together and involve neurological, metabolic, inflammatory and immunological circuits, and cognitive-emotional factors. Certain aspects of FM are still controversial, including pathophysiology. The current hypothesis suggests that FM mechanisms produce a hyperactivation of brain areas modulating pain, resulting in an altered pain perception. However, the mechanisms involved are still under debate, and the role of “neuro-inflamation” and the immune system is currently under study [1]. Widespread pain, fatigue, and insomnia are the main symptoms. Quality of life is significantly impacted. The current prevalence of FM is estimated to be over 2.45% in Spain and 2.64% in Europe, being far more predominant in women, at a rate of 95–96% [2,3,4]. CFS, on the other hand, has various causes and affects people from all socioeconomic groups and of all ages, although it is most common in the 30–50 age range. The estimated prevalence in the United States is 0.42%, with 70% of patients being women [5]. There are no updated data on incidence in Europe; however, it is estimated to currently affect around 2 million people [6]. Thus, both syndromes have a significant sanitary and socioeconomic impact [3,7]. People with FM also often present CFS, which is underdiagnosed [8]. 

Useful prevention and management strategies must be identified to face the major impact on quality of life, as well as health and social costs. Current pharmacological treatments for FM do not improve symptomatology as effectively as expected. In recent years, multidisciplinary non-pharmacological therapies have led to promising improvements in pain and quality of life [9,10]. These interventions are based mainly on cognitive-behavioral therapy, physical activity, health education, and pain neuroscience education [9,10]. However, nutrition and chronobiology have scarcely been considered despite their potential [11].

The evidence on the impact of nutrition and chronobiology on various aspects of binomial health–disease is relevant. Similarly, the evidence of its association with FM and CFS is rapidly growing [12,13,14,15,16]. 

FM is associated with oxidative stress and mitochondrial, immunological, and cardiovascular autonomic dysfunction [17,18,19,20,21]. Furthermore, recent hypotheses concerning its pathophysiology include the role of inflammation, gut dysbiosis, neuroinflammation, and DNA methylation [22,23]. Nutrition and chronobiology play relevant roles in all these processes. Several studies have associated intake patterns, food intake, macronutrient intake, and micronutrient levels, as well as weight and body composition, with the diagnosis and severity of FM [24,25,26,27]. However, more research is needed, and specific studies and recommendations are still lacking [28,29,30].

Additionally, the importance of certain nutritional deficiencies and low levels of certain micronutrients, amino acids, essential fatty acids, and coenzymes has also been associated with the severity of CFS symptoms [31]. 

Circadian rhythms are particularly relevant in metabolism, hormonal regulation, and cellular repair mechanisms. Altered rhythms have been linked to several diseases, such as neurodegenerative diseases, cancer, and cardiovascular disease, among others [32,33]. CFS is associated with aspects of chronobiology, including sleep quality, that seem to play a relevant role in the development and progression of the [13,14,15]. Recent studies also show the impact and relevance of chronobiology in FM [34]. A significant correlation was observed between the total scores of the Biological Rhythms Interview of Assessment in Neuropsychiatry (BRIAN) questionnaire, which evaluates the disruption of circadian rhythms, and higher levels of pain and worse functionality results in greater depression and worse quality of sleep. Furthermore, nutrition, chronobiology, and physical activity are deeply interrelated. 

**Figure 1:**
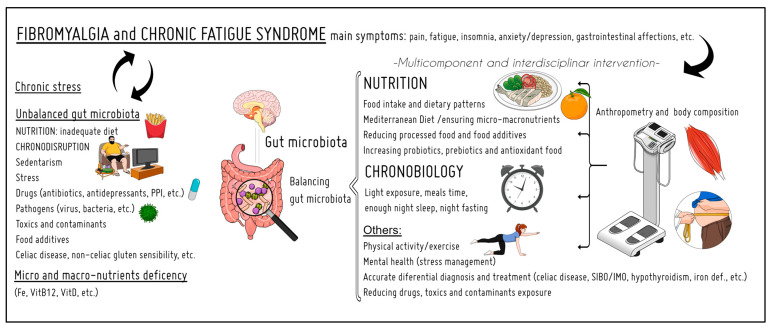
Summary of the topics addressed in this review. The relevance of nutrition and chronobiology in the prevention and treatment of fibromyalgia and chronic fatigue syndrome. PPI: proton-pump inhibitor; SIBO: small intestine bacterial overgrowth; IMO: intestinal methanogenic overgrowth.

Therefore, this narrative review offers a deep dive into the evidence of these two components—nutrition and chronobiology—in FM and CFS (and related aspects such as body composition, gut microbiota, and supplementation) to evaluate their importance and interconnection in prevention and treatment to improve quality of life (Figure 1).

## 2. Methods

A non-systematic narrative review of the literature available on healthy lifestyle strategies including nutrition and chronobiology to prevent and manage FM and CFS was conducted. The most relevant original scientific papers, clinical trials, meta-analyses, and reviews on these topics published before October 2023 in English were considered. Case reports and letters were excluded. The following keywords alone or in combination were included: diet, dietary habits, food intake, body composition, nutrition, nutrients, vitamins, minerals, vitamin D, iron, ferritin, vitamin B12, probiotics, gut microbiota, gut permeability, deficiency, deficit, supplementation, multidisciplinary approach, circadian rhythms, chronobiology, gut microbiota, dysbiosis, fibromyalgia, chronic fatigue syndrome, myalgic encephalomyelitis, and central sensitization.

The authors reviewed the full text of 156 papers to provide a critical discussion. PubMed, Scopus, and Web of Science were used as electronic database sources.

## 3. Results

### 3.1. Nutrition

#### 3.1.1. Dietary Patterns, Macronutrient Levels, and Nutritional Quality

Several studies have shown that the food intake of people with FM differs from healthy controls. Caloric intake and the mean consumption of nutrients, such as carbohydrates, protein, lipids, and several micronutrients, are lower in people with FM [27,35]. FM patients also presented alterations in their intake of different food groups, such as a lower consumption of dairy, bread, whole-grain cereals, fresh fruit, and fish [24]. Similarly, López-Rodriguez et al. (2017) [36] observed patterns of food avoidance related to lactose, vegetables, cereals, and caffeine in women with FM. However, FM patients had a higher intake of cured meat and sweetened beverages than healthy controls [12]. This, together with the intake of other unhealthy, nutrient-empty foods, could be related to higher rates of emotional eating in FM, likely mediated by hedonistic pathways [37]. A review by Rossi et al. (2015) [38] also reported more eating disorders in FM patients, including binge eating disorder (BED) and nocturnal eating. The latter may be associated with chronobiological disruption [39], as will be discussed later.

These findings are interesting if we consider that a higher percentage of protein intake is associated with a higher pain threshold in FM [27]. Moreover, a nearly daily intake of fruit and vegetables and a moderate intake of fish are associated with more promising psychosocial outcomes in women with FM [12]. 

Regarding CFS, few data are available. One study showed that 95% of the CFS group had poor fiber intake, and 70% had an unhealthy fat intake ([40,41]).

#### 3.1.2. Specific Diets

Many specific diets have been tested to evaluate their effect on FM symptoms, including high antioxidant and/or anti-inflammatory, elimination (glutamate, aspartame, lactose, histamine, and gluten), hypocaloric, vegan, vegetarian, lacto-ovo vegetarian, plant-based, raw vegan, and low fermentable oligo-, di-, and monosaccharides and polyols (FODMAP) diets [38,42,43]. 

Studies testing glutamate, aspartame, and histamine elimination diets showed no improvement in FM symptoms. However, the sample sizes were small, and the diets led to weight loss and improvement in digestive symptoms [44]. Thus, more studies are required to draw conclusions.

Clinical improvements in FM were observed after following various diets: (1) a diet rich in antioxidants, such as plant-based diets, (2) a low FODMAP diet, (3) a gluten-free diet, or (4) a low-calorie diet [38,42,43,45,46,47]. Da Silva et al. (2022) [45] studied the effect of a three-month nutritional intervention based on an anti-inflammatory diet (gluten-free, no sugar, no dairy, and no processed food) with a low FODMAP diet during the first month followed by the re-introduction of such foods. Functionality, pain, sleep quality using the Pittsburgh Sleep Quality Index (PSQI), and quality of life improved after the intervention, while no inflammatory markers changed [45]. 

This approach may be interesting for an FM subgroup with digestive comorbidities that alter microbiota, such as small intestine bacterial overgrowth (SIBO), intestinal methanogenic overgrowth (IMO), irritable bowel syndrome (IBS), celiac disease, food intolerances and allergies, and non-celiac gluten sensitivity, among others, that present with gut permeability and inflammation. Recent studies on non-celiac gluten sensitivity found the same prevalence in FM patients as in the general population [48]. However, non-celiac gluten sensitivity has a complex diagnosis. Moreover, it may be interesting to investigate other allergens such as beta-lactoglobulin or other proteins from cow’s milk and byproducts [49].

A lacto-ovo vegetarian dietary intervention alongside core stabilization exercises improved muscle mass and reduced body fat and pain in women with FM [50]. A Mediterranean diet enriched with walnuts also showed beneficial effects on fatigue, anxiety, depression, and eating disorders in women with FM [51]. 

The preliminary results of a 12-week program consisting of a variety of low-calorie diets (800 kcal) for persons with FM showed an improvement in symptoms at three weeks but no significant weight loss, which underscores that the effect was due to the calorie restriction itself and not weight loss [52]. The authors question the safety of such a restrictive approach and do not recommend this type of intervention. Furthermore, the observed improvement may be associated with the reduction in (potentially inflammatory) food intake.

Conditions such as obesity, food allergies, nutritional deficiencies, and food additive intake were revealed to be risk factors correlated with FM complications. Although no specific diet therapy has been identified for FM, a high-antioxidant diet, high-fiber foods such as fruits and vegetables, fewer processed foods, high-quality proteins, healthy fats, and nutritional supplementation, when required, are beneficial in alleviating FM symptoms [53]. As for CFS, few data were found on specific diets. 

#### 3.1.3. Micronutrient Levels in FM

Micronutrient deficiencies and low levels of certain vitamins and minerals have been observed in FM patients, including iron (low levels of ferritin), vitamin B12, and vitamin D [25,27,54]. Ferritin, vitamin B12, and vitamin D levels were negatively correlated with the number of tender points [54]. Furthermore, ferritin and vitamin B12 levels were independent risk factors that potentially play a role in the complex and are not fully understood in relation to the etiopathogenesis of FM [54]. Iron deficiency anemia and thalassemia minor are more prevalent in FM than in the general population [38,55,56], which indicates that iron deficiency is a risk factor for FM in young people and adults [55,57]. Furthermore, non-anemic iron deficiency (<50 ng/mL) has been observed in FM [25]. Iron plays an important role in metabolism. It is a co-factor for serotonin and dopamine production among other relevant functions (e.g., thyroid hormone production) and, therefore, might play a role in the etiology of FM [38].

Furthermore, people with FM showed a lower intake of vitamin C [27]. This, together with the increased excretion of vitamin C and K induced by the analgesics often prescribed in FM, increases the risk of iron deficiency [38].

Vitamin B12 is an independent risk factor in the etiopathogenesis of FM [54]. Vitamin B12 deficiency (<350–400 ng/dL) was observed in 42.4% of FM patients, who presented greater fatigue and memory loss than patients with higher levels, indicating that a vitamin B12 deficiency is significantly associated with fatigue in FM [58].

Several studies have found that patients with FM had lower levels of vitamin D than healthy controls [59]. A low level of vitamin D has been linked to fatigue, widespread muscle pain, and weakness [59,60]. Furthermore, various studies have suggested a connection between vitamin D deficiency and higher pain sensitivity in FM [61]. Although some authors suggest that vitamin D be considered in FM management, there are contrasting data regarding the benefits of supplementation on FM symptoms [38]. While there is a clear relationship between vitamin D deficiency and FM in a large subset of patients, it is difficult to establish this as the primary causation [62]. Serum vitamin D deficiency is associated with higher levels of serum proinflammatory cytokines (IL-6 and IL-8), and higher levels of serum proinflammatory cytokines are associated with a greater FM impact [63]. While no linkage was established between vitamin K levels and FM, positive correlations were found between IL-6 and the Fibromyalgia Impact Questionnaire (FIQ), and between TNF-alpha and physical role difficulty. This suggests that low-intensity inflammation may accompany FM and negatively impact physical activity [64]. Furthermore, an interesting association between vitamin D and iron status was observed in elite female athletes, which deserves further exploration [65].

FM patients had a lower intake of vitamins A, E, folate, selenium, calcium, zinc (Zn), and magnesium (Mg) [27]. FM has been associated with high levels of oxidative stress and lower antioxidant capacity, which may be linked to low levels of Mg and selenium (Se), among other micronutrients [38]. Antioxidant vitamins and minerals could reduce oxidative stress and improve the sensation of pain [27,66]. The intake of antioxidants from legumes, vegetables, and nuts was lower in people with FM; meanwhile, they consumed more antioxidants from grains and potatoes than the healthy population [66]. Their intake of omega-6 fatty acids was also lower.

Higher vitamin E consumption was correlated with increased vasodilation and better functionality in FM [27]. Vasodilation may improve vasomotor pain associated with vasomotor dysregulation and muscle hypoperfusion described in FM [27].

Low dietary intake of phosphorus, iron, Zn, group B vitamins, folate, and vitamin D has been linked to higher Fibromyalgia Impact Questionnaire Revised Version (FIQR) scores and reduced functionality [67]. Greater vitamin B6 intake was associated with lower pain intensity using the pain Visual Analog Scale (VAS), which shows the relevance of micronutrients in symptom and pain severity in women with FM [67]. A deficiency of vitamin D, iron, Mg, Zn, calcium, and manganese (Mn) in FM was also detected by Rezende et al. (2019) [61].

Patients with FM have significantly lower plasma concentrations of the three branched amino acids (BCAAs), namely, valine, leucine, isoleucine, and phenylalanine, than normal controls [38,68], which is likely associated with lower absorption or intake, since protein intake is lower in FM patients [27,35,38,69]. BCAAs supply energy to the muscles and regulate muscle protein synthesis, potentially playing a role in the etiopathogenesis of FM [69]. Regarding amino acids, a subgroup of FM patients presented altered tryptophan metabolism, suggesting a tryptophan deficiency [70]. This could also be associated with gut microbiota dysbiosis.

Bjorklund et al. (2018) [16] concluded that when optimal levels of nutrients are achieved, pain levels usually decrease. This study also underscored the harmful effects of heavy metals such as mercury, cadmium, and lead, which increase oxidative stress and interfere with the availability of essential nutrients [16]. Furthermore, muscle pain may be associated with nutrient imbalances, such as a deficiency of amino acids, Mg, Se, group B vitamins (especially vitamin B12), iron, and vitamin D. In this regard, micronutrient levels have been studied in relation to several metals that cause oxidative stress. Plasma levels of lipid peroxidation, protein carbonyls, nitric oxide, copper (Cu), Mn, and aluminum were significantly higher, and Mg and Zn were significantly lower in FM patients. Oxidative stress parameters, FIQR, and tender points were positively associated with Cu, aluminum, and Mn. A significant negative association was observed between Zn and Mg, and FIQR, tender points, and oxidative stress parameters [71]. Thus, heavy metals such as aluminum induce oxidative stress parameters and decrease the levels of essential trace elements such as Mg and Zn, which may be responsible for the severity of FM [71].

Women with FM had a lower dietary intake of Mg and Ca; however, serum levels for these nutrients did not differ between groups. A low dietary intake of minerals was correlated with worsened pain threshold parameters [72]. A higher dietary intake of Mg and tryptophan through walnuts decreased fatigue, anxiety, and depression [51]. However, these improvements may also be linked to other nutrients in walnuts. Regarding Mg, it is worth noting that only 0.8% of Mg is found in the blood (of which 0.3% is in the serum), with the rest being in the soft tissues, muscles, and bones [73]. Due to this distribution of Mg, its concentration in one area, such as the serum, may not accurately reflect Mg status throughout the whole body. Therefore, determining the Mg status of the whole body is a challenge. Moreover, due to the narrow range of Mg in the blood that is easily maintained through the exchangeable Mg pool, blood Mg levels can be misleading and mask a deficient Mg body status [74].

#### 3.1.4. Micronutrient Levels in CFS

In an analytical observational study, mean serum 25-OH vitamin D levels were moderately to severely suboptimal in 60.1% of CFS participants not taking supplements. This finding was lower over winter/spring and summer/autumn compared to the general population [41,75].

Regarding fatty acids, a significant reduction was shown in the CFS cohort for the omega-3 (*n*-3) to omega-6 polyunsaturated (*n*-6) fatty acid (PUFA) ratio and the eicosapentaenoic acid (EPA) to arachidonic acid (AA) ratio [41,76]. A statistically significant positive relationship was also observed between the *n*-3 to n-6 PUFA ratio and serum Zn, an indicator of the inflammatory response. There was also a positive relationship between the severity of CFS symptoms and certain *n*-6 PUFA, *n*-9 PUFA, and oleic and palmitic acid, as well as a negative relationship between the *n*-3 to *n*-6 ratio and the severity of CFS symptoms [41,76].

The importance of certain nutritional deficiencies such as vitamin C, the vitamin B complex, sodium, Mg, Zn, folic acid, L-carnitine, L-tryptophan, essential fatty acids, and coenzyme Q10 has also been highlighted in the severity of CFS symptoms [16].

Mean serum phosphate concentration was found to be significantly lower in the CFS cohort but still within the reference range in an analytical observational study [41,77]. In one study, 14% of participants with CFS met the diagnostic criteria for phosphate diabetes yet did not show a significant difference in symptoms when compared to those with CFS without a phosphate diabetes diagnosis [41,77].

### 3.2. Anthropometric Parameters and Body Composition

Obesity and overweight are common conditions in FM and are more prevalent in FM than in healthy controls. Increased weight is linked to dietary habits, limited physical activity, eating disorders such as BED or nocturnal eating, poor sleep, and hypometabolism via thyroid dysfunction [24,38]. Obesity and overweightness in FM are also associated with depression, anxiety, and fatigue, among other symptoms [38,43]. There is a correlation between the symptom severity as measured by FIQR and body mass index (BMI) [78]. A higher BMI has been associated with higher dysfunctionality and lower quality of life in FM [38]. Accordingly, Marum et al. (2017) [42] studied a nutritionally balanced approach for weight loss based on a low FODMAP diet for four weeks. The preliminary results showed a reduction in BMI, weight, waist circumference, pain, and severity of FM [42] but no impact on body composition. However, in a recent study, body fat was positively correlated with the widespread pain index, and muscle was negatively correlated with the FIQ questionnaire score, suggesting that body composition should represent a basic element in the clinical approach of patients with FM [79]. Other studies have found that body fat percentage is higher in people with FM than in the healthy population [80], and higher muscle mass is associated with better psychological health, less pain, and a lower impact of FM symptoms [50], thus stressing the relevance of body composition in FM outcomes. However, a recent study identified a significant reduction in muscle function (dynapenia) in FM patients without any loss of muscle mass [81]. Muscle strength can be recorded using the handgrip strength test and physical performance with the Short Physical Performance Battery [82].

Obese women with FM had higher blood pressure, pulse, pain intensity, and worse results on the Fibromyalgia Impact Questionnaire. As for biochemistry, higher concentrations of inflammatory proteins such as IL-6 were observed in obese patients with FM. Significantly decreased blood flow and an increased concentration of pyruvate were detected in obese patients compared with non-obese patients. There was a significant correlation between inflammatory proteins and sedentary behavior and health status in obese patients with FM. Metabolism and inflammation interact in female patients with FM with obesity and might cause chronic low-grade inflammation [83]. Furthermore, FM status and depressive symptoms are linked to BMI and physical performance in mid to late life [84]. Screening for obesity and the monitoring of BMI changes should be considered in the treatment of patients with FM, as well as to monitor body composition and muscle functionality [50,81].

### 3.3. Gut Microbiota

#### 3.3.1. Gut Microbiota in FM

Altered microbiome composition was observed in individuals with FM [85]. Dysbiosis, or the alteration of gut microbiota, predisposes patients to pathologies such as inflammatory bowel disease, obesity, autoimmune and neurodegenerative diseases, and FM, among many others [86]. Intestinal dysbiosis and the associated abnormal secretion of hormones and vitamins such as cortisol, serotonin, vitamin D, and thyroid hormones have been observed in FM patients [86]. An alteration in the circadian cycle with the consequent sleep disorder, fatigue, and weakness is triggered by an altered hormone concentration [86] and vice versa. Furthermore, hormone concentrations affect the communication of the microbiota with the brain (gut microbiota–brain axis) that takes place through the vagus nerve and modulates the hypothalamic–pituitary–adrenal axis. In the complex pathogenesis of FM, an autonomic dysfunction has been identified [87,88]. The link between the alteration of microbiota and FM has been studied, establishing a close connection between the neuroenteric and central nervous systems [86]. Increased gut permeability and gut inflammation are associated with autonomic dysfunction in FM and CFS, respectively [49].

Pimentel et al. (2004) [89] showed that 100% of their FM study group presented SIBO. In this study, the degree of somatic pain was significantly correlated with the hydrogen breath test level [86,89]. When SIBO was eradicated, patients experienced noteworthy clinical improvement [86,89]. This would explain why the low FODMAP diet generally indicated for SIBO and IBS treatment improved FM symptoms [42,45]. SIBO is highly prevalent in IBS patients, with up to 78% presenting SIBO [90,91]. Certain functional digestive disorders such as abdominal pain, functional bloating or gas, IBS mixed-type, and IBS constipation-type appear to be more prevalent in adults with FM [92]. Low FODMAP diets significantly improved IBS symptoms [93], as well as SIBO [94]. The presence of SIBO was detected in 33.8% of persons with gastroenterological complaints who underwent a breath test and is significantly associated with smoking, bloating, abdominal pain, and anemia [95]. SIBO and other processes affecting gut microbiota permeability and functionality are likely underdiagnosed prevalent conditions in FM and should undergo further study. 

Alterations have been observed in the composition and function of the gut microbiome in women with FM, including changes in the relative abundance of certain bile acid-metabolizing bacteria. Bile acids can affect various physiological processes, including visceral pain [96]. The alterations in bile acid metabolizing bacteria were accompanied by significant alterations in the serum concentration of secondary bile acids, including a marked depletion of α-muricholic acid. Serum α-muricholic acid was highly correlated with symptom severity, including pain intensity and fatigue. This circulating bile acid alteration in FM is potentially secondary to upstream gut microbiome alterations [96]. 

Another recent study showed that the diversity of the microbiome in the FM group was lower than in the control group, and the reduced stool propionate levels (a short-chain fatty acid) may be associated with the decreased abundance of propionate-producing bacteria [97]. In contrast, another study showed that neither the composition nor the alpha or beta diversity of the fecal microbiome differed from healthy controls, suggesting that the contribution of the gut microbiome to the pathophysiology of FM is limited [98].

Thus, various digestive disorders associated with gut microbiota imbalance and gut permeability such as celiac disease, SIBO, IBS, non-celiac gluten sensitivity, food intolerances and allergies, *Helicobacter pylori*, etc., could play a role in FM symptomatology. Hypothyroidism, stress, insomnia, an unhealthy diet (rich in high saturated fat and sugar), and sedentarism, which are frequent conditions in FM, may be behind digestive disorders contributing to gut microbiota imbalance. Drugs, especially antibiotics, chronic proton-pump inhibitor (PPI) treatment, opioids, and non-steroidal anti-inflammatory drugs, among others, play a documented role in gut microbiota imbalance [99,100,101]. Furthermore, gut microbiota may be altered in response to antidepressants [102]. Antidepressants are often prescribed in FM since depression is one of the main comorbidities, and some antidepressants reduce pain and increase functionality [103]. 

#### 3.3.2. Gut Microbiota in CFS

*Faecalibacterium prausnitzii* and *Eubacterium rectale* are abundant, health-promoting butyrate producers in the human gut and were found in reduced quantities in persons with ME/CFS. A deficient microbial capacity for butyrate synthesis was also confirmed. The abundance of *F. prausnitzii* was inversely associated with fatigue severity. The functional nature of gut dysbiosis, with its underlying microbial network disturbance, was observed in CFS [104]. 

As for the pathophysiology of IBS, which is frequent in FM and CFS, abnormal intestinal bacterial profiles and low bacterial diversity appear to play important roles. The intestinal abundance of *Alistipes* spp., *F. prausnitzii, Eubacterium biforme, Holdemanella biformis, Prevotella* spp., *Bacteroides stercoris, Parabacteroides johnsonii, Bacteroides zoogleoformans*, and *Lactobacillus sppnine* increased after fecal microbiota transplantation in patients with IBS. These increases were inversely correlated with IBS symptoms and fatigue severity. The intestinal abundance of *Streptococcus thermophilus* and *Coprobacillus cateniformis* decreased in patients with IBS after fecal microbiota transplantation and was correlated with the severity of IBS symptoms and fatigue. Several of these bacteria produce short-chain fatty acids (SCFAs), especially butyrate, that modulate the immune response and intestine hypersensitivity and decrease intestinal permeability and motility. Probiotics containing the identified bacteria, as well as protein-rich diets, could increase the intestinal abundance of *Alistipes*, and a plant-rich diet could increase the intestinal abundance of *Prevotella* spp. to improve IBS and fatigue [105].

Another recent study describes microbial and metabolomic dysbiosis in CFS patients. Short-term CFS patients showed significant microbial dysbiosis, while long-term patients had largely resolved microbial dysbiosis but had metabolic and clinical aberrations. These revealed potential functional mechanisms underlying disease onset and duration, including reduced microbial butyrate biosynthesis and a reduction in plasma butyrate, bile acids, and benzoate [106].

Food contaminants and other toxins also alter and negatively affect the gut microbiota balance [107,108]. The evidence suggests that suspected gut permeability or gut microbiota alteration should be carefully studied, as should the potential original cause.

### 3.4. Supplementation

#### 3.4.1. Supplementation in FM

Although the role of dietary supplements remains controversial, the results of clinical trials with vitamin D, Mg, iron, vitamin B12, and probiotics supplementation are promising [47,54]. However, supplementation cannot be systematic or generalized for FM but must be studied individually, considering comorbidity, symptomatology, and micronutrient deficiencies. Investigating the cause of the deficiency and searching for a solution is the mid-to-long-term objective.

As for vitamin D, high-dose supplementation in young people improved short-term musculoskeletal pain and long-term functional capacity [109]. In elderly subjects, musculoskeletal pain and long-term quality of life improved [109]. However, another study found no differences in pain following vitamin D supplementation for 12 weeks [110]. A recent review on vitamin D supplementation in FM and widespread chronic pain concluded that there is quality evidence demonstrating that an appropriate supplementation may have beneficial effects on patients with an established vitamin D blood deficiency. It also reported that vitamin D supplementation leads to pain reduction [111], suggesting that it might also alleviate the pain associated with FM and widespread chronic pain, especially in vitamin D-deficient individuals [111]. Vitamin D supplementation also had significant positive effects on physical function (measured by FIQ), role limitations due to emotional health, social function, and general health, but not on scores of the pain VAS or Beck’s Depression Inventory (BDI) [112]. Supplementation improves pain caused by FM; however, there is a gap regarding specific recommendations in healthcare systems [113].

Regarding iron, ferric carboxymaltosa supplementation yielded greater improvements after 42 days in functionality (FIQR total score), Brief Pain Inventory (BPI) total score, fatigue visual numeric scale (VNS) score, and iron indices in iron-deficient patients (<50 mg/L) with FM [29]. Ensuring adequate vitamin C intake or vitamin C supplementation helps improve iron indices. Low iron and vitamin D levels are also associated, but the mechanism is not currently understood [65]. It may be interesting to improve both levels simultaneously if low.

In a recent study, it was observed that functionality (FIQR scores) in all domains in people with FM improved significantly after treatment with daily sublingual vitamin B12. Vitamin B12 also improved anxiety scores. The authors postulate that vitamin B12 has a strong potential to be considered at least an adjunctive therapy in FM [114]. Frequent injections of high-concentrate vitamin B12 combined with individual daily doses of oral folic acid may result in blood saturations high enough to provide relief in a subgroup of patients with FM and/or CFS (Regland et al., 2015). However, these patients may be taking opioid analgesics and other drugs that need to be demethylated as part of their metabolism, which could represent a risk of counteracting interference with vitamin B12 and acid folic supplementation [26]. The efficacy of mecobalamin (vitamin B12) supplementation in women with FM is currently undergoing further study [115].

Regarding Mg, and considering its antinociceptive action as an *N*-methyl-d-aspartate (NMDA) receptor antagonist, it can prevent central sensitization and attenuate preexisting pain hypersensitivity. NMDA receptors play a role in pain transduction, making it the object of research in relation to various pain conditions [116]. The beneficial effects of Mg therapy have been reported in FM, as well as in patients with neuropathic pain, such as malignancy-related neurologic symptoms, diabetic neuropathy, postherpetic neuralgia, and chemotherapy-induced peripheral neuropathy, dysmenorrhea, headaches, and acute migraine attacks [116]. Along these lines, some authors suggest that dietary Mg and/or Mg supplements be considered to maintain an optimal Mg balance all through life to help prevent oxidative stress and chronic conditions associated with aging [117]. A recent study concluded that Mg intake was inversely correlated with *C*-reactive Protein (CRP) in the FM group [118]. Furthermore, daily supplementation of oral Mg chloride improved mild/moderate stress and reduced pain in FM patients [119]. However, larger clinical trials are required.

The use of probiotics to treat FM has been studied and the results are positive and promising; however, more research is required to expand our knowledge of the benefits of different species and strains [120]. Probiotic supplementation may be of special interest in digestive pathologies with alterations in intestinal microbiota. As already mentioned, microbiota is often altered in FM. Microorganisms in the gut regulate brain processes through the gut microbiota–brain axis, affecting pain, depression, and sleep quality. Consequently, prebiotics and probiotics may potentially improve physical, psychological, and cognitive health in FM patients with an altered microbiota balance. In a small preliminary study, probiotic supplementation significantly improved sleep quality, depression, anxiety, and pain scores in FM patients, while prebiotic supplementation significantly improved pain scores and sleep quality [121]. Symbiotic nutritional supplements can improve dysregulated immune-neuroendocrine interaction involving inflammatory and stress responses in women diagnosed with FM, particularly when there is no prior CFS diagnosis, as well as their perceived levels of stress, anxiety, depression, and quality of life [122]. Moreover, multispecies probiotic supplementation improved impulsivity and decision-making in persons with FM [123]. However, more studies are needed.

Coenzyme Q10 (CoQ10) is a key element in mitochondrial bioenergetics, and its antioxidant role has been researched thoroughly. Low CoQ10 levels are associated with many degenerative and chronic disorders including FM [38,124]. CoQ10 supplementation improves clinical parameters, including pain and fatigue, depression, and quality of life scores in FM [38,46,125,126,127,128].

Melatonin is a pineal hormone with a complex role. It is linked to sleep and inflammatory, oxidative, and immunological processes, leading it to be studied in FM as well. A recent review investigated the use of melatonin supplementation in rheumatological diseases. It showed positive results for melatonin administration in FM, osteoarthritis, and osteoporosis/osteopenia but not in rheumatoid arthritis and lupus. Melatonin supplementation was well tolerated with mild side effects [129].

An orally administered amino acid-based supplement containing blended L-lysine, L-arginine, oxo-proline, *N*-acetyl-l-cysteine, L-glutamine, and *Schizonepeta tenuifolia* over 24 weeks showed sustained augmentation of IGF-1, which could improve clinical symptoms, including stress-related weight gain, and stress-associated lower-than-normal hGH levels in individuals with FM [130].

Furthermore, supplementation with chlorella green algae, acetyl-l-carnitine, or a combination of vitamin C and E, as well as tryptophan (5-HTP) and S-adenosyl-L-methionine, also yielded improved pain measures [38,46,125].

Supplementing ongoing pregabalin and duloxetine treatment with palmitoylethanolamide (PEA) and acetyl-L-carnitine (ALC) for 24 weeks in FM patients was effective. It improved scores on the widespread pain index (WPI) and FIQR [131].

However, more well-designed experimental research with more participants is required to reinforce current data on the efficacy of supplements in FM. On the other hand, when mild micronutrient deficiency is detected, improving dietary intake or dietary intervention is always recommended as the first treatment option. Supplementation should always be personalized, supervised, and managed by the appropriate health professional.

#### 3.4.2. Supplementation in CFS

Supplementation with coenzyme CoQ10 improved FM-associated fatigue [132]. In the same line, oral CoQ10 plus NADH supplementation improved fatigue and biochemical parameters in CFS patients after 8 weeks of treatment [133]. Furthermore, a recent review suggests the capability of coenzyme Q10, L-carnitine, Zn, methionine, nicotinamide adenine dinucleotide (NAD), and vitamins C, D, and B supplementation in decreasing fatigue in FM and SCF [134]. On the other hand, D-ribose and omega-3 fatty acid supplementation led to positive outcomes for CFS symptom relief [41,135]. Furthermore, a multivitamin and mineral supplementation assayed in persons with CFS showed improvements in before-and-after measurements of superoxide dismutase (SOD) antioxidant enzyme activity. Various fatigue measures improved, including fatigue itself, sleep disorder, autonomic nervous system symptoms, headaches, and subjective feelings surrounding infection. Some quality-of-life measures were also statistically significant, including total physical function, physical role, pain, mental vitality, and mental health function scores [41,136].

Mg supplementation improved fatigue at six months of treatment but led to no significant relationship at two years of treatment [41,135]. 

Regarding probiotic supplementation, few data are available for CFS, despite the large number of associated microbiota studies. A small cohort study reported subjective improvement post-trial and improvement in neurocognitive function measures but found no significant changes in fatigue or physical activity scores. No major changes occurred in the gastrointestinal microflora [137]. One randomized clinical trial detected a decrease in several systemic inflammatory markers like CRP and TNF-α in FM/CFS patients after an oral intake of *Bifidobacterium infantis* [138]. Another non-controlled pilot study with 13 ME/CFS patients showed improvements in well-being and oxidative and inflammatory parameters after probiotic intake [139]. Current evidence on probiotic treatment for CFS is low because of the low quality of studies. Clinical studies on the efficacy of probiotics in ME/CFS patients are currently ongoing [140,141].

Regarding microbiota modulation, in recent studies on mice models, 16S rDNA sequencing analysis indicated that Astragalus polysaccharide (APS), an important bioactive component derived from the dry root of *Astragalus membranaceus*, regulated the abundance of gut microbiota and increased production of SCFAs and anti-inflammatory bacteria. Additionally, APS reversed the abnormal expression of Nrf2, NF-κB, and their downstream factors in the brain–gut axis and alleviated the reduction in SCFAs in the cecal content associated with CFS. APS also modulated the changes in serum metabolic pathways induced by CFS. Lastly, it was verified that butyrate exerted antioxidant and anti-inflammatory effects on neuronal cells. In short, APS may increase the SCFA content by regulating the gut microbiota and SCFAs (especially butyrate) and may further regulate oxidative stress and inflammation in the brain, thus alleviating CFS [142].

### 3.5. Chronobiology

#### 3.5.1. Chronobiology and FM

Poor sleep quality is one of the core symptoms of FM and is significantly correlated with the severity of pain, fatigue, depression, and stress symptoms and reduced quality of life, especially regarding mental health. This suggests that treatment of this disease should include sleep disorder interventions [143]. A significant correlation was observed between the total scores of the BRIAN questionnaire and higher levels of pain, worse functionality, greater depression, and worse quality of sleep [34].

Disruptions in circadian variations of melatonin, cytokines, and serotonin levels, which are fully regulated by circadian rhythm, are observed in FM. Patients had lower melatonin secretion during the hours of darkness than healthy subjects, potentially associated with impaired sleep at night, daytime fatigue, and altered pain perception. Furthermore, elevated evening serum cortisol was also observed in FM patients, as well as higher cytokine levels. Thus, the circadian rhythm is a relevant aspect to be studied to elucidate FM pathogenesis, diagnosis, and treatment [144]. The impact of night-shift work on health is under study and is associated with increased pain perception, among other things [145]. Night-shift workers are exposed to nocturnal light and are more prone to circadian rhythm disorders. A meta-analysis showed that night-shift work is associated with the suppression of melatonin production, especially among fixed night-shift workers [146].

In line with sleep disorders, a recent review concluded that due to the clinical and scientific relevance of the insomnia–pain–anxiety pathological cycle, and given the impact it has on FM, it is especially important to develop programs for FM patients based mainly on improving sleep quality [147].

Chronic pain and circadian rhythms have also been studied using actigraphy data and machine learning algorithms, among other mechanisms. Chronic pain predictions were more accurate using rest–activity rhythm features than sleep or activity features. The intraday variability of rest–activity rhythm was the most predictive feature, with elevated values in pain associated with disturbed sleep. Rest–activity rhythms can effectively detect subjects with chronic pain [148].

Furthermore, nociceptive, neuropathic, central, and mixed pain states showed a circadian pattern of pain. FM pain, as well as postoperative pain, trigeminal neuralgia, and migraines, were associated with higher pain scores in the morning. Moreover, temporomandibular joint pain, neuropathic pain, labor pain, biliary colic, and cluster headaches increased throughout the day, peaking in the evening or night. However, arthritis and cancer pain did not show any circadian rhythmicity. The circadian rhythm of pain was not found to be altered in patients on analgesics [149].

Circadian light exposition is also relevant in FM. One study examined the effect of one daily hour of bright morning light treatment (active treatment) compared to a dim morning light treatment (comparison treatment) over four weeks. Both the bright and dim light treatment groups achieved significant and similar levels of improvement in pain intensity, pain interference, physical function, depressive symptoms, and sleep disturbance. Clinical improvement in the FIQR score was observed, comparable to results reported after following physical exercise treatments, and minimal side effects were observed [150].

Night fasting, a reduced window of daytime intake, and the intake of two to three meals/day in the first part of the day, including breakfast, contribute to the regulation of circadian clocks [151,152]. 

All these results suggest that recommendations for synchronizing circadian rhythms are key for health.

#### 3.5.2. Chronobiology and CFS

The evidence of autonomic dysfunction in CFS is abundant. However, little is known about its association with circadian rhythms and endothelial dysfunction. The amplitude and stability of daily activity rhythm, measured with an actigraph, were lower in CFS patients than in controls. Distal skin temperature was sensitive to environmental temperature and showed lower nocturnal values in CFS patients than in controls (only in winter). A post-lunch dip in activity and a peak in distal skin temperature occurred in controls but not in CFS patients. These findings suggest that circadian temperature regulation and skin vasodilator responses may play a role in CFS [13].

One study explored autonomic responses through an orthostatic test and analysis of the peripheral skin temperature variations and vascular endothelium state in CFS patients. The results suggest that CFS patients showed modifications in circadian rhythm and hemodynamic measures, which were associated with endothelial biomarker levels (ET-1 and VCAM-1). Dysautonomia and vascular tone abnormalities need to be further studied to provide potential therapeutic targets for CFS [14].

The results of a meta-analysis demonstrate that sleep is altered in CFS, with changes seeming to differ between adolescents and adults, suggesting sympathetic and parasympathetic nervous system alterations in ME/CFS [153].

## 4. Conclusions and Future Perspectives

This literature review summarizes the evidence on the association of dietary habits and diet, food intake, macronutrient and micronutrient intake, and body composition (especially skeletal muscle mass) with pain intensity, physical functionality, fatigue, psychological outcomes, digestive health, and quality of life in persons with FM and CFS. Of particular interest is a Mediterranean diet that ensures appropriate protein intake at main meals, based on legumes, fish, and eggs, that is rich in antioxidants and fiber such as vegetables and fruits, ancient whole grains, probiotic foods, healthy fats such as nuts and extra virgin olive oil, and that avoids/reduces sugar, saturated fat, and red and processed meat and foods [12,47,50,51,53]. Limiting ultra-processed food and certain food conservatives and additives that can negatively affect gut microbiota is also an interesting approach [154]. Several studies have pointed out that increasing antioxidant intake, improving digestive symptoms, and preventing or rebalancing deficiencies in certain micronutrients can improve FM symptoms [22,28,29,30,45]. Micronutrient deficiencies need to be regularly monitored, especially ferritin, vitamin D, and vitamin B12. Insufficient caloric intake or altered intake patterns must also be identified and the causes studied (anxiety, depression, eating disorders, digestive issues, body perception, restriction diets, BED, nocturnal eating, etc.). Group and personalized nutrition education and diet therapy led by a registered dietitian is a low-cost approach with interesting benefits. Although more specific studies and recommendations are still lacking, future studies, protocols, and guidelines should consider including them. 

Moreover, diet, chronobiology, physical exercise, and general lifestyle can contribute to the modulation of gut microbiota, which has been demonstrated to show differences in FM and CFS compared to a healthy population [155]. Unhealthy gut microbiota can affect macronutrient digestion and mineral and vitamin absorption (iron, Mg, vitamin D, etc.), vitamin biosynthesis (e.g., vitamin B12), and hormone and neurotransmitter secretion (cortisol, serotonin, thyroid hormones, etc.), all of which are often altered in FM and CFS. Such alterations can have a significant impact on fatigue, pain, anxiety, and depression. Relevant health-related gut microbiota metabolites such as SCFAs, like butyrate, and secondary biliary acids may be also altered. This can affect gut sensitivity and permeability, immune response, and redox equilibrium and contributes to low-grade inflammation, among other issues. Furthermore, SCFAs and secondary biliary acids play a role in mitochondrial energy metabolism and oxidative stress management. Microbiota status should be examined thoroughly in FM and CFS to identify potential dysbiosis, gut permeability, and associated SIBO, IMO, or other conditions, etc., as well as to identify the root cause of the dysbiosis (celiac disease, hypothyroidism, drugs, pollutants, food allergies, ultra-processed food intake, etc.) and form an integral approach to both as required (pharmacological treatment, diet therapy, supplementation, etc.). New clinical protocols to properly address differential diagnosis in FM and CFS (hypothyroidism, celiac disease, iron depletion myopathy, etc.), including gut permeability and dysbiosis, and a gut microbiota approach are urgently required. SIBO and IMO are likely underdiagnosed but prevalent conditions in FM and must be further studied. New research on gut microbiota and microbiome in FM and CFS could generate new therapeutic options including microbiota modulation with drugs, diet, probiotics, prebiotics, postbiotics, etc.

Sleep quality and circadian rhythms, which are key for gut microbiota health, pain, fatigue, and anxiety, are also altered in FM and CFS. The dysregulation of the circadian levels of hormones such as cortisol, melatonin, and serotonin has been associated with FM. Circadian rhythms disrupted by light at night and mistimed food intake alter hormonal rhythms and metabolism [39]. Likewise, the dysregulation of circadian distal body temperature variation and vascular endothelial state has also been associated with CFS. Thus, chronobiological aspects seem to play an important role in these conditions. Nutrition, gut microbiota, and chronobiology are deeply interrelated [156]. Adequate mealtimes and a minimum of 12 h of night fasting help regulate circadian clocks [151,152] and, therefore, improve insomnia and fatigue. Exposure to sunlight, especially in the morning, significantly helps regulate circadian rhythms. On the other hand, a balanced diet, together with good sleep quality and adequate exercise, contributes to improving body composition, increasing muscle mass, and thus improved metabolism, and health. 

For all these reasons, nutrition and chronobiology must be a relevant part of multicomponent interventions, alongside physical activity and stress management, to improve the quality of life of persons with FM and associated CFS. A summary of nutritional and chronobiologic recommendations based on the current revision results is provided in Table 1.

## Figures and Tables

**Table 1:** Summary of main nutritional and chronobiologic recommendations for persons affected by FM and FCS based on the current revision results.

Nutritional and Chronobiologic Recommendations
Nutrition ✓ Have a healthy food intake pattern following Harvard plate proportions of food groups and macronutrients ✓ Have high adherence to a healthy diet with high contents of vegetables (vitamins, fiber, antioxidants) and healthy fat (e.g., Mediterranean Diet) ✓ Include fermented foods (probiotics) ✓ Avoid/reduce simple sugars, alcohol, red and processed meat, processed and fast food, and food additives ✓ Ensure the appropriate intake of energy and micro- and macro-nutrients. Medical monitoring of micronutrient levels and supplementation when required ✓ Improve body composition: increase skeletal muscle mass and quality
Chronobiology ✓ Eat between 2 and 3 meals a day and do 12–14 h of night fasting ✓ Having lunch and dinner early is better than late, and the latter at least 3 h before going to sleep ✓ Follow regular mealtimes ✓ Exposure to outdoor light throughout the morning and afternoon, and keeping bedroom dark at night ✓ Avoid blue screen light of electronic devices in the evening and night ✓ Sleep 7–9 h/night and go to sleep early better than late
Others ✓ Reduce drug intake, toxins, and contaminant exposure, when possible ✓ Ensure adequate differential diagnosis and identification of comorbid conditions (celiac disease, non-celiac gluten sensibility, hypothyroidism, iron deficiency, B12 deficiency, vitD deficiency, SIBO/IMO, food allergy, and intolerances, etc.), as well as their appropriate treatment ✓ Have an active life and practice physical exercise ✓ Reduce and properly manage stress

## Data Availability

Not applicable.
